# Migration, invasion, and metastasis are mediated by *P-Rex1* in neuroblastoma

**DOI:** 10.3389/fonc.2024.1336031

**Published:** 2024-05-31

**Authors:** Jillian C. Jacobson, Jingbo Qiao, Elizabeth D. Cochran, Sullivan McCreery, Dai H. Chung

**Affiliations:** Division of Pediatric Surgery, Department of Surgery, University of Texas Southwestern Medical Center and Children’s Health, Dallas, TX, United States

**Keywords:** neuroblastoma, *P-Rex1*, migration, invasion, metastasis

## Abstract

Neuroblastoma accounts for approximately 15% of pediatric cancer-related deaths despite intensive multimodal therapy. This is due, in part, to high rates of metastatic disease at diagnosis and disease relapse. A better understanding of tumor biology of aggressive, pro-metastatic phenotypes is necessary to develop novel, more effective therapeutics against neuroblastoma. *Phosphatidylinositol 3,4,5-trisphosphate-dependent Rac exchanger 1 (P-Rex1)* has been found to stimulate migration, invasion, and metastasis in several adult malignancies. However, its role in neuroblastoma is currently unknown. In the present study, we found that P-Rex1 is upregulated in pro-metastatic murine models of neuroblastoma, as well as human neuroblastoma metastases. Correspondingly, silencing of *P-Rex1* was associated with decreased migration and invasion *in vitro*. This was associated with decreased AKT-mTOR and ERK2 activity, dysregulation of Rac, and diminished secretion of matrix metalloproteinases. Furthermore, increased *P-Rex1* expression was associated with inferior relapse-free and overall survival via tissue microarray and Kaplan-Meier survival analysis of a publicly available clinical database. Together, these findings suggest that *P-Rex1* may be a novel therapeutic target and potential prognostic factor in neuroblastoma.

## 1 Introduction

Neuroblastoma, the most common extracranial solid tumor in children, accounts for approximately 15% of pediatric cancer-related deaths (1, 2). This is largely due to the poor prognoses of patients with high-risk disease who, despite significantly morbid, intensive therapy, demonstrate overall survival rates of less than 40–50% (3–5). Approximately half of patients with neuroblastoma present with distant metastases at the time of diagnosis (4, 6–8). Even with aggressive multimodal therapeutic regimens, which include chemotherapy, radiation, stem cell transplant, surgical resection, and immunotherapy, patients with metastatic neuroblastoma continue to demonstrate poor prognoses (6, 9, 10). Therefore, further biological understanding of these aggressive, pro-metastatic phenotypes is necessary to help facilitate the development of novel, more effective therapies.

*Phosphatidylinositol 3,4,5-trisphosphate-dependent Rac exchanger 1 (P-Rex1)* is a Rac guanine nucleotide exchange factor (RacGEF) that is upregulated in numerous adult cancers, including breast cancer, prostate cancer, melanoma, and liver cancer (11–15). It is activated downstream of *phosphoinositide 3-kinase (PI3K)* and G-protein coupled receptors (GPCRs) to promote activation of *Rac* (12), which has been implicated in cell migration, a process that is critical for tumor metastasis (13). Although P-Rex1 demonstrates negligible protein expression in most benign tissues (13–15), it is thought to stimulate numerous aspects of oncogenesis, including migration, invasion, and metastasis in various malignancies (13, 15–18).

Despite several preclinical studies implicating *P-Rex1* in migration, invasion, and metastasis in various adult malignancies, its role in neuroblastoma, a lethal pediatric malignancy in which approximately half of affected children demonstrate metastatic disease at diagnosis, is currently unknown. Therefore, the purpose of this study was to evaluate the potential role of *P-Rex1* in neuroblastoma.

## 2 Materials and methods

### 2.1 Antibodies and reagents

Primary antibodies against P-Rex1 (#13168), phospho-AKT (#9271), AKT (#9272), phospho-mTOR (#2971), mTOR (#2972), phospho-p44/42 MAPK (ERK1/2) (#4370), p44/42 MAPK (ERK1/2) (#9102), and TIMP1 (#8946) were obtained from Cell Signaling Technology (Danvers, MA, USA). Primary antibody against β-actin was obtained from Sigma-Aldrich (St. Louis, MO, USA). Secondary anti-mouse, anti-rabbit, and anti-goat antibodies were obtained from Santa Cruz Biotechnology, Inc. (Santa Cruz, CA, USA).

### 2.2 Cell lines and culture

The human NB cell lines, including the *MYCN*-amplified BE(2)-M17, CHP-212, IMR-32, JF, LAN-1, SK-N-BE(2), SK-N-BE(2)-C (a clonal subline of SK-N-BE(2), hereafter referred to as BE(2)-C), SK-N-DZ, and the *MYCN* non-amplified SHEP, SK-N-AS, SK-N-SH, and SH-SY5Y, were purchased from the American Type Culture Collection (Manassas, VA). Cells were maintained in Rockwell Park Memorial Institute (RPMI) culture medium 1640 with 10% fetal bovine serum (FBS) at 37°C in a humidified atmosphere consisting of 5% CO_2_ and 95% air.

BE(2)-C and SK-N-SH cells were stably transfected to silence *P-Rex1* (BE(2)-C/shPREX1 and SK-N-SH/shPREX1, respectively) or control (BE(2)-C/shCON and SK-N-SH/shCON, respectively) utilizing shRNA plasmids as per the manufacturer’s instructions (sc-760230SH, Santa Cruz Biotechnology, Inc., Dallas, TX, USA). Puromycin (Thermo Fisher Scientific, Waltham, MA, USA) was used as a selection antibiotic.

JF cells were transiently transfected to silence *P-Rex1* (JF/siPREX1) or control (JF/siCON) utilizing siRNA as per the manufacturer’s instructions (sc-76023 and sc-37007 (respectively), Santa Cruz Biotechnology, Inc., Dallas, TX, USA).

### 2.3 Animal studies

**Figure 1:**
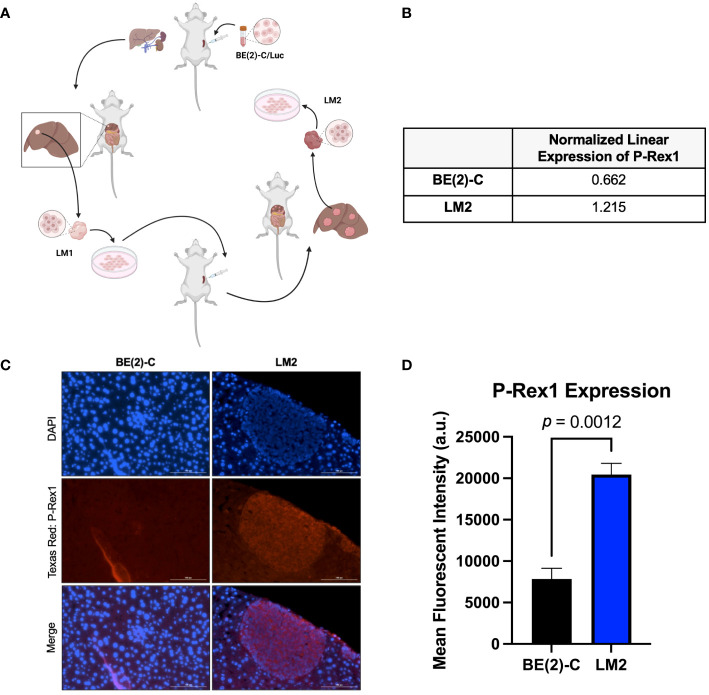
P-Rex1 was upregulated in pro-metastatic murine models of neuroblastoma. **(A)** Schematic representation of the *in vivo* selection model utilized to isolate pro-metastatic, aggressive subclones of neuroblastoma cells from foci of murine hepatic metastases. Image created with BioRender.com. **(B)** The pro-metastatic subclone, LM2, demonstrated increased normalized linear expression of P-Rex1 on reverse phase protein array analysis relative to the parental BE(2)-C cell line. **(C)** Representative images demonstrating expression of P-Rex1 in murine hepatic metastases produced after splenic injection of BE(2)-C (left) or LM2 (right) cells using immunohistochemistry. The scale bar represents 100 μm. **(D)** Murine hepatic metastases produced after splenic injection of the pro-metastatic subclone, LM2, demonstrated increased expression of P-Rex1, relative to murine hepatic metastases produced after splenic injection of the parental cell line, BE(2)-C (*p* = 0.0012; mean ± SEM.).

4–6-week-old male athymic nude mice were maintained as previously described (19). All studies were approved by the Institutional Animal Care and Use Committee at Vanderbilt University Medical Center (Protocol M1500059–00) and were conducted in accordance with guidelines issued by the National Institutes of Health (20). Serial splenic injection of BE(2)-C cells was performed as previously described to generate a pro-metastatic subclone of neuroblastoma (BE(2)-C/LM2, hereafter referred to as LM2) (**Figure 1A**). At sacrifice, liver metastases were fixed in formalin for further evaluation. This metastatic model was chosen to recapitulate the theoretical mechanism of tumor spread seen in human patients with metastasis originating from a primary tumor (as opposed to a tail vein injection model) and due to its essentially 100% frequency of metastatic spread within one month.

### 2.4 Reverse-phase protein array

Reverse-phase protein array (RPPA) analysis was performed at the MD Anderson Cancer Center Functional Proteomics Core Facility as previously described (21–24). Briefly, cellular proteins from BE(2)-C parental cells and LM2 subclones were denatured and serially diluted in five two-fold dilutions with lysis buffer. Lysates were arrayed on nitrocellulose-coated slides using an Aushon Biosystems 2470 Arrayer (Aushon BioSystems, Billerica, MA, USA). Slides were probed with approximately 500 validated primary antibodies plus a corresponding biotin-conjugated secondary antibody. Obtained signals were amplified and subsequently visualized using 3,3’-Diaminobenzidine (DAB) colorimetric reactions. Slides were scanned, analyzed, and quantified using Array-Pro Analyzer software (Media Cybernetics, Inc., Rockville, Maryland, USA) to generate spot intensity. Each dilution curve was fitted with a logistic model. Protein concentration data were then normalized for protein loading. Correction factors were calculated by median-centering across samples and antibodies. Protein expression was compared using normalized linear values.

### 2.5 Immunohistochemistry and immunofluorescence staining

Immunohistochemistry (IHC) staining was performed using the DAKO EnVision+ System-HRP from Dako North America, Inc. (Carpinteria, CA). Mouse neuroblastoma xenografts were excised and fixed in 10% buffered formalin overnight and embedded in paraffin wax. Tumor sections (5 μm) were mounted on glass slides. Samples were deparaffinized and rehydrated. The antigen was retrieved using 0.01 M sodium-citrate buffer (pH 6.0) at a sub-boiling temperature for 25 minutes after boiling in a microwave oven. To block endogenous peroxidase activity, the sections were incubated using the Dako kit at 4°C for 15 minutes. Sections were subsequently washed with dH_2_O twice and then permeabilized with 3% BSA/PBS with Triton twice for ten minutes each. Sections were blocked for 60 minutes in the dark prior to incubation with primary antibody against P-Rex1 at 4°C overnight (HPA001927, Sigma-Aldrich, St. Louis, MO, USA). They were then washed with buffer four times for 5 minutes each and incubated with Alexa Fluor 647 secondary antibody for 60 minutes at room temperature. Sections were again washed in buffer and the reaction was terminated by immersing slides in dH_2_O. Coverslips were mounted and slides were left to dry. DAPI was used for staining nuclei. Images were captured using a Cytation 5 Cell Imaging Multi-Mode reader (Agilent, Santa Clara, CA, USA).

### 2.6 Immunohistochemistry analysis of human neuroblastomas

IRB exemption from the UT Southwestern Human Research Protection Program was received. IHC was performed using standard techniques on de-identified human neuroblastoma samples from four patients to evaluate the expression of P-Rex1 in primary tumors as well as sites of invasion and metastasis. P-Rex1 expression was compared between the primary tumor and sites of metastasis within the same patient to mitigate potential confounding introduced by patient-specific differences in expression. Given that bone marrow biopsy is a typical component of neuroblastoma staging at our institution, these were the most readily available archived pre-treatment samples. All samples had been formalin-fixed, paraffin-embedded, and archived with informed consent by the UT Southwestern/Children’s Medical Center Dallas Department of Pathology for research purposes.

### 2.7 Immunoblotting

Cells were collected using cell lysis buffer, and denatured protein samples were prepared for immunoblotting as we have previously described (25). Equal amounts of protein were loaded and separated by NuPAGE 4–12% Bis-Tris gel, followed by transfer onto PVDF membranes (Bio-Rad, Hercules, CA, USA). Membranes were blocked with 5% nonfat milk in TBS-T for 1 hour at room temperature. The blots were then incubated with antibodies against the human target proteins by using rabbit or mouse anti-human antibodies (1:500–2000 dilution) overnight at 4°C. Anti-rabbit or anti-mouse secondary antibodies conjugated with HRP were incubated for 1 hour and visualized using an enhanced chemiluminescence detection system (PerkinElmer, Waltham, MA, USA). Densitometry was used to assess quantitative protein expression using ImageJ software (Rasband, W.S., ImageJ, U.S. National Institutes of Health, Bethesda, Maryland, USA, https://imagej.nih.gov/ij/, 1997–2018).

### 2.8 Migration assays

Neuroblastoma cells were serum-starved for 18 hours. Subsequently, 5 x 10^4^ cells (BE(2)-C/shCON or BE(2)-C/shPREX1; JF/siCON or JF/siPREX1) in 500 μL of serum-free media were added to the upper chamber of each insert. 750 μL of complete media (RPMI with 10% FBS) were added to the lower chambers. Cell invasion chambers were incubated at 37°C, 5% CO_2_ atmosphere for up to 24 (JF cells) or 48 hours (BE(2)-C cells). Then, the media in the upper chamber was aspirated and any remaining media was gently removed with a cotton-tipped applicator, taking care not to damage the bottom membrane of the Transwell insert. Migrated cells were fixed in 0.5% paraformaldehyde in PBS for 30 minutes and subsequently stained with 0.1% crystal violet dye for 10 minutes prior to air drying. Cells were viewed with a Biotek Cytation 5 Cell Imaging Multi-Mode Reader (Agilent, Santa Clara, CA, USA) and counted.

### 2.9 Invasion assays

Invasion assays were performed in a similar manner as the migration assays. However, for the invasion assays, 2 x 10^5^ transfected JF cells in 500 μL of serum-free media were added to the upper chamber of each insert. Furthermore, Transwell inserts were coated with Corning^®^ Matrigel^®^ Basement Membrane Matrix Growth Factor Reduced (Catalog #354230, Lot #3073002) prior to adding neuroblastoma cells. The Matrigel was diluted to 300 μg/mL in coating buffer as per the manufacturer’s instructions. 0.1 mL of diluted Matrigel matrix coating solution was added to each Transwell^®^ insert (Corning Product #3422) and incubated at 37°C for two hours to facilitate polymerization. Both BE(2)-C and JF cells were incubated for 48 hours prior to staining. The remainder of the protocol was identical to that of the migration assays.

Percent invasion rates were calculated by mean number of cells that had invaded through the Corning Matrigel matrix-coated permeable support membrane divided by the mean number of cells that had migrated through the uncoated permeable support membrane times 100 (26).

### 2.10 Wound healing/scratch assay

To evaluate the effects of *P-Rex1* silencing on cell migration *in vitro*, confluent monolayers of SK-N-SH/shCON and SK-N-SH/shPREX1 cells tagged with green fluorescent protein (GFP) in 6-well plates were scraped with 200 μl pipette tips. Cells were incubated and wound closure was observed microscopically for up to 96 hours after scraping. Cells were photographed using the Biotek Cytation 5 Cell Imaging Multi-Mode Reader (Agilent, Santa Clara, CA, USA).

### 2.11 Cell proliferation and cytotoxicity assay

BE(2)-C/shCON and BE(2)-C/shPREX1 cells, JF/siCON and JF/siPREX1 cells, and SK-N-SH/shCON and SK-N-SH/shPREX1 cells were plated in 96-well plates in triplicate at 500, 5000, and 2000 cells per well, respectively, in RPMI culture medium with 10% FBS. Cell viability was measured using Cell Counting Kit-8 (CCK-8) colorimetric assay (Dojindo Molecular Technologies, Inc., Rockville, MD, USA) and counted using the Cytation 5 Cell Imaging Multi-Mode reader (OD 450 nm) (Agilent, Santa Clara, CA, USA) every 24 hours for up to 96 hours after plating. CCK-8 allows sensitive colorimetric assays for determination of cell viability in cell proliferation and cytotoxicity assays, where the amount of dye generated by the activities of dehydrogenases in the cells is directly proportional to the number of living cells.

### 2.12 Clonogenic assay

BE(2)-C/shCON and BE(2)-C/shPREX1 cells were plated at 1000 cells per well in a 6-well plate in triplicate. Cells were cultured for 14 days. Colonies were stained with 0.02% crystal violet dye, photographed, and counted using the Bio-Rad Gel Doc XR+ Imager (Bio-Rad, Hercules, CA, USA).

### 2.13 Patient database

R2 (http://r2.amc.nl), a publicly available genomics analysis and visualization platform (27) was utilized to interrogate microarray and RNA-seq data of human tumors from the Kocak (n = 649 samples; accessible at: https://www.ncbi.nlm.nih.gov/geo/query/acc.cgi?acc=GSE45547) (28) and Versteeg (n = 88 samples) neuroblastoma databases. These databases were used to investigate *P-Rex1* expression and possible associations with *Rac 1, Rac 2, Rac 3, ERK1, ERK 2, MMP-2, and MMP-9* expression in human samples, as well as patient outcomes as a function of *P-Rex1* expression, respectively.

### 2.14 RAC GTPase assay

2 x 10^6^ cells were plated in a 10 cm plate and allowed to attach overnight. Cells were serum-starved for 6 hours prior to overnight stimulation with serum-supplemented media. Rac1 activity was subsequently assessed using the Active Rac1 Detection Kit (Cell Signaling Technology, Danvers, MA, USA) as per the manufacturer’s instructions with one exception: cell lysates were incubated at 4°C overnight as opposed to one hour to facilitate interaction with and binding to PAK1 p21-binding domain (PBD) beads. Anti-Rac3 antibody was purchased from Abcam (Abcam, Cambridge, UK). To briefly summarize, protein lysates were collected and prepared for each cell group. Agarose beads were resuspended by swirling the glutathione resin. The resin slurry was added to a tube, centrifuged, and washed. GST-PAK1-PBD was added to the glutathione resin. Cell lysates were then also added. Tubes were vortexed and subsequently incubated overnight at 4°C with gentle agitation. Tubes were centrifuged, the supernatant was aspirated, and the resin was washed. After the final wash, the tube was centrifuged, the supernatant aspirated, and reducing buffer was added to the resin. Samples were vortexed and incubated at room temperature for two minutes. The tubes were again centrifuged and the resin was discarded prior to eluting the samples.

Rac2 activity was similarly assessed using the Rac2 Activation Assay Kit (Abcam, Cambridge, UK) as per the manufacturer’s instructions with one exception: cell lysates were incubated at 4°C overnight as opposed to one hour. In brief, protein lysate was collected and prepared for each cell group. Protein lysates were resuspended in a PAK1 PBD agarose bead slurry. Tubes containing protein lysates with the PAK1 PBD agarose beads were vortexed and incubated overnight at 4°C with gentle agitation. Tubes were then centrifuged, and the supernatant was aspirated. The bead pellet was washed three times, centrifuging after each wash. After removal of the supernatant, the bead pellet was resuspended in reducing buffer and boiled.

GTPγS and GDP controls were also performed as per manufacturer’s instructions for each set of experiments. Immunoblotting was then performed as previously described.

### 2.15 Enzyme-linked immunosorbent assay

BE(2)-C/shCON and BE(2)-C/shPREX1 cells were plated at 4 x 10^6^ cells per well in 6-well plates. Media was collected 24 hours after plating. MMP-2 and MMP-9 secretion were assessed using R&D Systems Quantikine ELISA kits (R&D Systems, Inc., Minneapolis, MN, USA) as per the manufacturer’s instructions.

### 2.16 Statistical and experimental analysis

All experiments were repeated in triplicate. The scoring index and relative expression values were expressed as mean ± SEM. Statistical analyses were performed using Student’s and Welch’s *t*-tests, Mann-Whitney U tests, and analyses of variance using GraphPad Prism (version 9.4.1, GraphPad Software, San Diego, California USA, www.graphpad.com). A *p* value ≤ 0.05 was considered significant.

## 3 Results

### 3.1 Pro-metastatic murine models of neuroblastoma demonstrated upregulation of P-Rex1

Reverse-phase protein array analysis showed a 1.8-fold increase in P-Rex1 expression between the BE(2)-C parental and the pro-2metastatic LM2 subclone cells (0.662 vs. 1.215 a.u., respectively) (**Figure 1B**).

IHC analysis of P-Rex1 expression was performed on hepatic metastatic foci produced after splenic injection of BE(2)-C vs. LM2 cells. Mean fluorescent intensity was calculated as peak fluorescence minus background fluorescence. Hepatic metastases from splenic injection of LM2 cells demonstrated significantly higher P-Rex1 expression when compared to hepatic metastases from splenic injection of BE(2)-C cells (20452.3 ± 1347.5 a.u. vs. 7849.0 ± 1290.5 a.u., respectively, *p* = 0.0012) (**Figures 1C, D**).

### 3.2 P-Rex1 was upregulated in human neuroblastoma metastases

**Figure 2:**
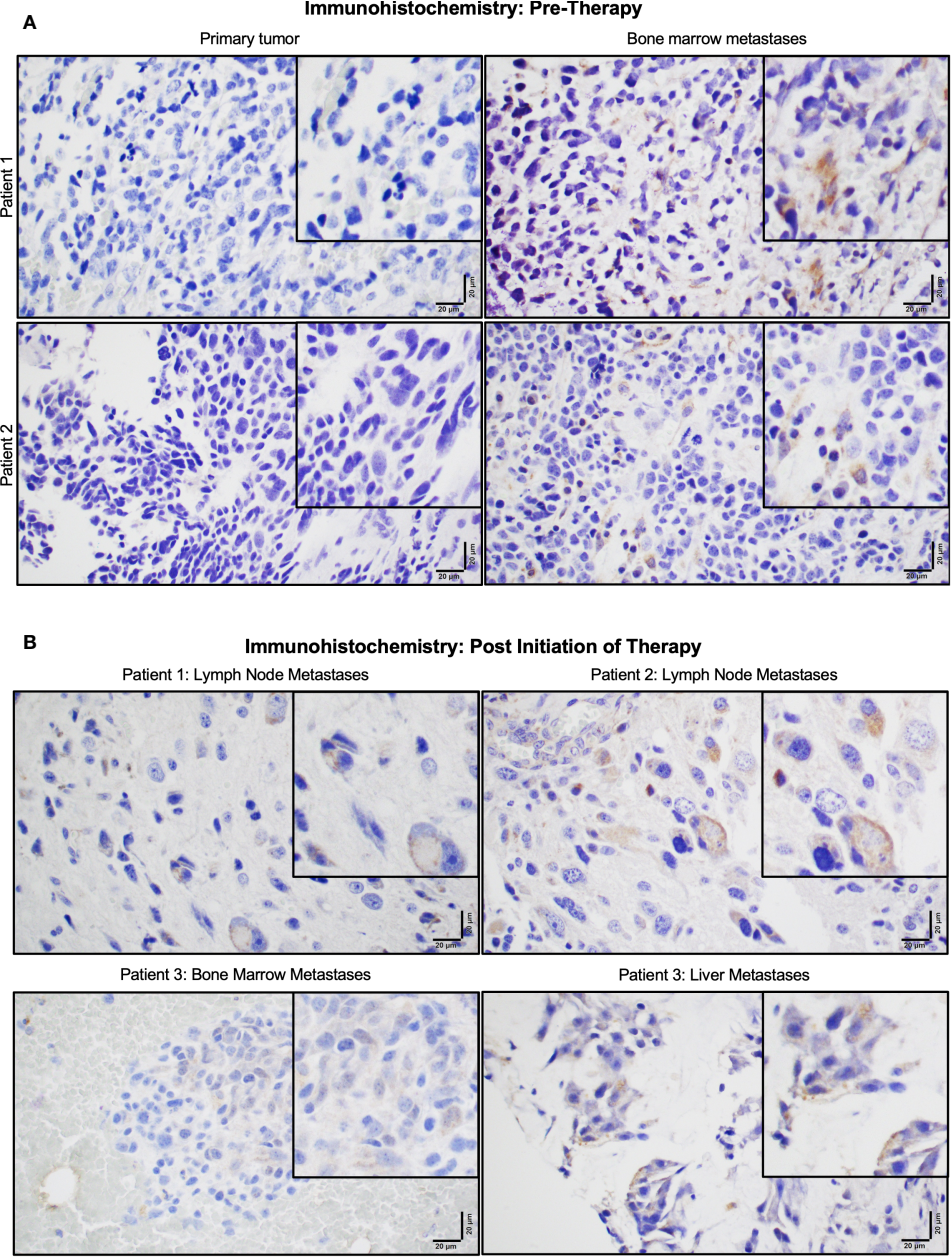
P-Rex1 was upregulated in human neuroblastoma metastases. **(A)** Patients demonstrated negligible expression of P-Rex1 in their primary tumor and increased P-Rex1 expression in the cytoplasm of neuroblastoma cells at sites of bone marrow metastases prior to initiation of therapy. **(B)** After systemic therapy, patients demonstrated persistent expression of P-Rex1 in metastases of the lymph nodes (Patient 1: top left, Patient 2: top right), bone marrow (Patient 3: bottom left), and liver (Patient 3: bottom right) after treatment. The scale bar represents 20 μm.

P-Rex1 expression was compared between primary tumors and paired bone marrow metastases using IHC in archived samples of four neuroblastoma patients obtained prior to initiation of therapy. All four patients demonstrated negligible expression of P-Rex1 in their primary tumor and increased P-Rex1 expression in the cytoplasm of neuroblastoma cells at sites of bone marrow metastases. Representative images from two patients are shown in **Figure 2A**.

Three of the four patients also had archived neuroblastoma tissue samples obtained from sites of metastases following systemic therapy (**Figure 2B**). All three patients demonstrated persistent expression of P-Rex1 in metastatic lesions from the lymph nodes, bone marrow, and liver after treatment. These findings prompted further evaluation of the role of *P-Rex1* in neuroblastoma and its potential role in driving migration, invasion, and metastasis.

### 3.3 P-Rex1 was ubiquitously expressed in parental human neuroblastoma cell lines

**Figure 3:**
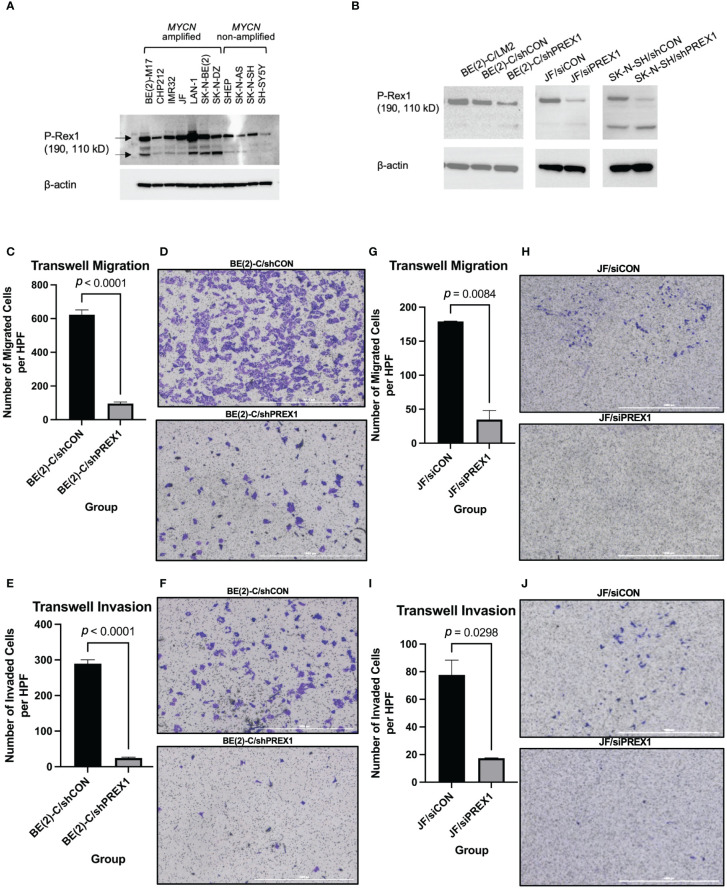
Silencing of *P-Rex1* decreased migration and invasion of neuroblastoma cells. **(A)** P-Rex1 is ubiquitously expressed across eleven neuroblastoma cell lines regardless of *MYCN* amplification status, as demonstrated by immunoblotting. **(B)** Successful transfection stably silencing P-Rex1 was confirmed using immunoblotting demonstrating decreased protein expression of P-Rex1 by the BE(2)-C/shPREX1 cell line relative to control (BE(2)-C/shCON). Correspondingly, the LM2 pro-metastatic subclone demonstrated increased protein expression of P-Rex1 relative to control. Successful transfection transiently silencing P-Rex1 in JF cells and stably silencing P-Rex1 in SK-N-SH cells was also confirmed using immunoblotting demonstrating decreased protein expression of P-Rex1 by the silencing cell lines relative to control. **(C)** Silencing of *P-Rex1* resulted in decreased Transwell migration in BE(2)-C cells. **(D)** Representative images of Transwell migration of BE(2)-C/shCON cells (top) versus BE(2)-C/shPREX1 cells (bottom). The scale bar represents 1000 μm. **(E)** Silencing of *P-Rex1* resulted in decreased Transwell invasion in BE(2)-C cells. **(F)** Representative images of Transwell invasion of BE(2)-C/shCON cells (top) versus BE(2)-C/shPREX1 cells (bottom). The scale bar represents 1000 μm. **(G)** Silencing of *P-Rex1* resulted in decreased Transwell migration in JF cells. **(H)** Representative images of Transwell migration of JF/siCON cells (top) versus JF/siPREX1 cells (bottom). The scale bar represents 1000 μm. **(I)** Silencing of *P-Rex1* resulted in decreased Transwell invasion in JF cells. **(J)** Representative images of Transwell invasion of JF/siCON cells (top) versus JF/siPREX1 cells (bottom). The scale bar represents 1000 μm.

To determine that P-Rex1 was a generalizable target in neuroblastoma, immunoblotting was performed using 11 human neuroblastoma cell lines, including the *MYCN*-amplified BE(2)-M17, CHP-212, IMR-32, JF, LAN-1, SK-N-BE(2), and SK-N-DZ, and the *MYCN* non-amplified SHEP, SK-N-AS, SK-N-SH, and SH-SY5Y cell lines. P-Rex1 protein expression was ubiquitously found in all 11 cell lines, irrespective of *MYCN* amplification status (**Figure 3A**).

The BE(2)-C cell line was then stably transfected to silence *P-Rex1* in order to determine functional and mechanistic insights. Efficient transfection was confirmed using immunoblotting demonstrating decreased protein expression of P-Rex1 by the BE(2)-C/shPREX1 cell line relative to control (BE(2)-C/shCON) (**Figure 3B**). Correspondingly, the LM2 pro-metastatic subclone demonstrated increased protein expression of P-Rex1 relative to control. The JF and SK-N-SH cell lines were also transiently and stably transfected, respectively, to silence *P-Rex1*. Efficient transfection was confirmed using immunoblotting demonstrating decreased protein expression of P-Rex1 relative to control (**Figure 3B**).

### 3.4 Silencing of *P-Rex1* was associated with decreased cell migration and invasion of neuroblastoma cells

Silencing of *P-Rex1* resulted in decreased Transwell migration (BE(2)-C/shCON: 622.8 ± 28.93 cells/hpf vs. BE(2)-C/shPREX1: 95.56 ± 9.30 cells/hpf, *p* < 0.0001) (**Figures 3C, D**) and invasion (BE(2)-C/shCON: 289.5 ± 10.90 cells/hpf vs. BE(2)-C/shPREX1: 24.43 ± 2.29 cells/hpf, *p* < 0.0001) (**Figures 3E, F**) of neuroblastoma cells *in vitro* after 48 hours of incubation. These data corresponded to a 46.49% invasion rate for the BE(2)-C/shCON cell line versus a 25.56% invasion rate for the BE(2)-C/shPREX1 cell line, further demonstrating the decreased potential for invasion associated with silencing of *P-Rex1*.

Silencing of *P-Rex1* resulted in decreased Transwell migration (JF/siCON: 179.0 ± 0.58 cells/hpf vs. JF/siPREX1: 34.7 ± 13.38 cells/hpf, *p* = 0.0084) (**Figures 3G, H**) of neuroblastoma cells *in vitro* after 24 hours of incubation. Silencing of *P-Rex1* resulted in decreased Transwell invasion (JF/siCON: 77.7 ± 10.68 cells/hpf vs. JF/siPREX1: 17.3 ± 0.33 cells/hpf, *p* = 0.0298) (**Figures 3I, J**) of neuroblastoma cells *in vitro* after 48 hours of incubation.

Furthermore, staining of JF/siCON and JF/siPREX1 cells after 96 hours of incubation demonstrated colony formation of invaded JF/siCON cells and the absence of colony formation of JF/siPREX1 cells, further demonstrating inhibition of invasion associated with silencing of *P-Rex1* (**Supplementary Figure 1**).

### 3.5 Silencing of *P-Rex1* inhibited cellular migration of SK-N-SH neuroblastoma cells

**Figure 4:**
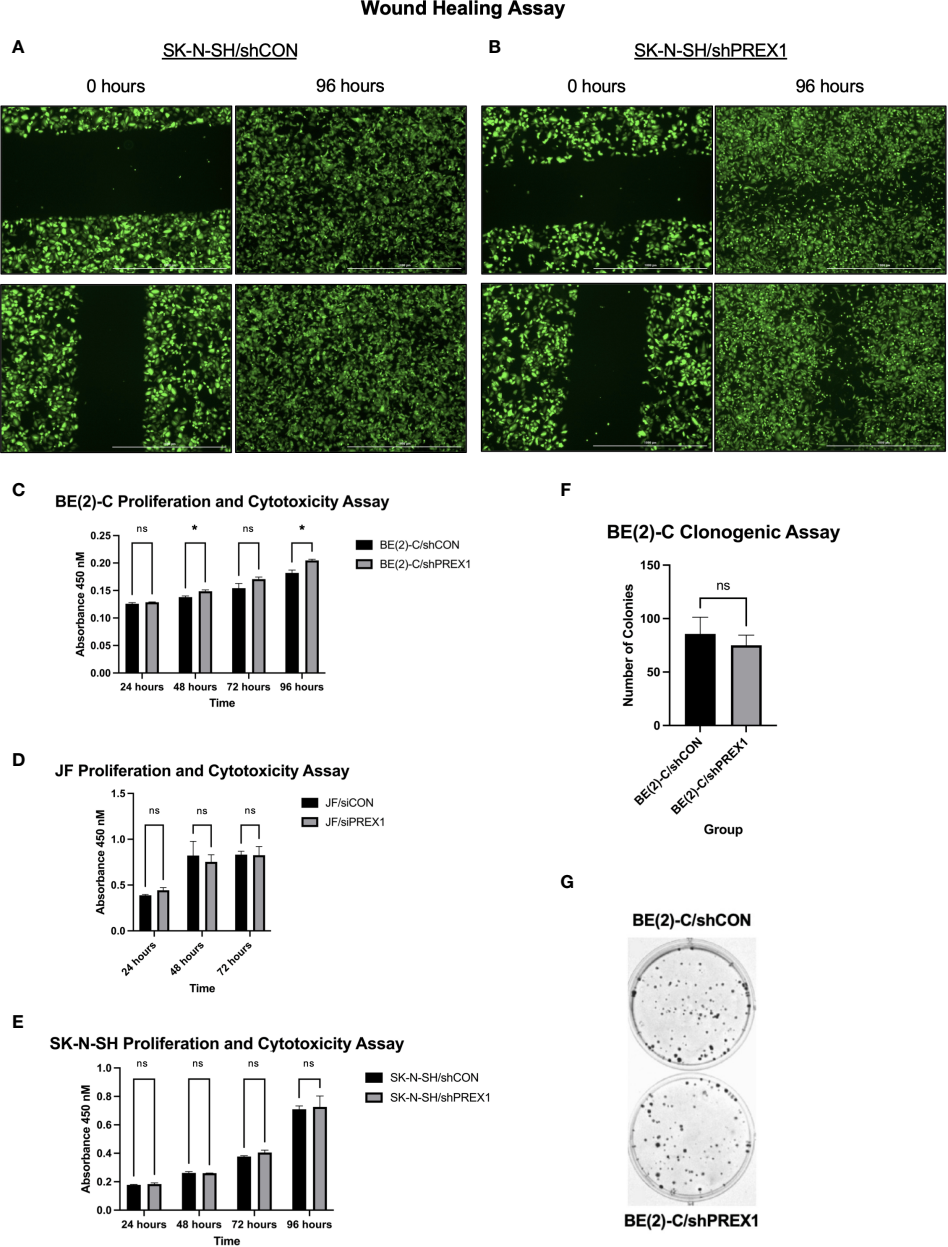
Silencing of *P-Rex1* decreased migration and wound healing of neuroblastoma cells but did not affect viability or colony formation *in vitro*. **(A)** SK-N-SH/shCON cells demonstrated complete closure of their wound gap at 96 hours. **(B)** SK-N-SH/shPREX1 cells continued to demonstrate a wound gap at 96 hours. **(C)** BE(2)-C/shPREX1 cells failed to demonstrate any significant decrease in cell viability, as evaluated by CCK-8 proliferation and cytotoxicity assays performed 24, 48, 72, and 96 hours after plating. **(D)** JF/siPREX1 cells failed to demonstrate any significant decrease in cell viability, as evaluated by CCK-8 proliferation and cytotoxicity assays performed 24, 48, and 72 hours after plating. **(E)** SK-N-SH/shPREX1 cells failed to demonstrate any significant decrease in cell viability, as evaluated by CCK-8 proliferation and cytotoxicity assays performed 24, 48, 72, and 96 hours after plating. **(F)** There was no significant difference in colony formation between control and silencing groups, as demonstrated by a clonogenic assay. **(G)** Representative images of colony formation of BE(2)-C/shCON cells (top) versus BE(2)-C/shPREX1 cells (bottom). (mean ± SEM; ns, not significant; *p < 0.05).

Wound healing assays were performed to assess the effect of *P-Rex1* silencing on cellular migration and wound healing in SK-N-SH neuroblastoma cells. Representative images are shown in **Figures 4A, B**. SK-N-SH/shCON cells demonstrated complete closure of the wound 96 hours after scratching (**Figure 4A**), whereas SK-N-SH/shPREX1 cells continued to demonstrate a wound gap (**Figure 4B**).

### 3.6 Silencing of *P-Rex1* did not affect cell viability or colony formation

Inhibition of cell migration was independent of changes in cell viability (**Figures 4C–E**). BE(2)-C/shPREX1 cells failed to demonstrate any significant decrease in cell viability, as evaluated by CCK-8 proliferation and cytotoxicity assays performed 24, 48, 72, and 96 hours after plating (absorbance at: 24 hours (shCON: 0.1260 ± 0.0020 vs. shPREX1: 0.1287 ± 0.0006 a.u., *p* = 0.138), 48 hours: (shCON: 0.1380 ± 0.0020 vs. shPREX1: 0.1487 ± 0.0025 a.u., *p* = 0.005), 72 hours: (shCON: 0.1543 ± 0.0084 vs. shPREX1: 0.1707 ± 0.0038 a.u., *p* = 0.060), 96 hours: (shCON: 0.1820 ± 0.0052 vs. shPREX1: 0.2047 ± 0.0021 a.u., *p* = 0.009) (**Figure 4C**).

JF/siPREX1 cells failed to demonstrate any significant decrease in cell viability, as evaluated by CCK-8 proliferation and cytotoxicity assays performed 24, 48, and 72 hours after plating (absorbance at: 24 hours (siCON: 0.3877 ± 0.0092 vs. siPREX1: 0.4437 ± 0.0280 a.u., *p* = 0.062), 48 hours: (siCON: 0.8217 ± 0.1156 vs. siPREX1: 0.7527 ± 0.0784 a.u., *p* = 0.543), 72 hours: (siCON: 0.8317 ± 0.0380 vs. siPREX1: 0.8250 ± 0.0953 a.u., *p* = 0.918) (**Figure 4D**).

SK-N-SH/shPREX1 cells also failed to demonstrate any significant decrease in cell viability, as evaluated by CCK-8 proliferation and cytotoxicity assays performed 24, 48, 72, and 96 hours after plating (absorbance at: 24 hours (shCON: 0.1780 ± 0.0030 vs. shPREX1: 0.1837 ± 0.0080 a.u., *p* = 0.3479), 48 hours: (shCON: 0.2620 ± 0.0096 vs. shPREX1: 0.2600 ± 0.0010 a.u., *p* = 0.754), 72 hours: (shCON: 0.3770 ± 0.0056 vs. shPREX1: 0.4050 ± 0.0168 a.u., *p* = 0.090), 96 hours: (shCON: 0.7093 ± 0.0235 vs. shPREX1: 0.7263 ± 0.0764 a.u., *p* = 0.743) (**Figure 4E**).

There was also no significant difference in colony formation between BE(2)-C groups, as demonstrated by a clonogenic assay (BE(2)-C/shCON: 85.67 ± 9.025 colonies vs. BE(2)-C/shPREX1: 75.00 ± 5.508 colonies, *p* = 0.381) (**Figures 4F, G**).

### 3.7 *P-Rex1* regulated activation of the AKT-mTOR pathway

**Figure 5:**
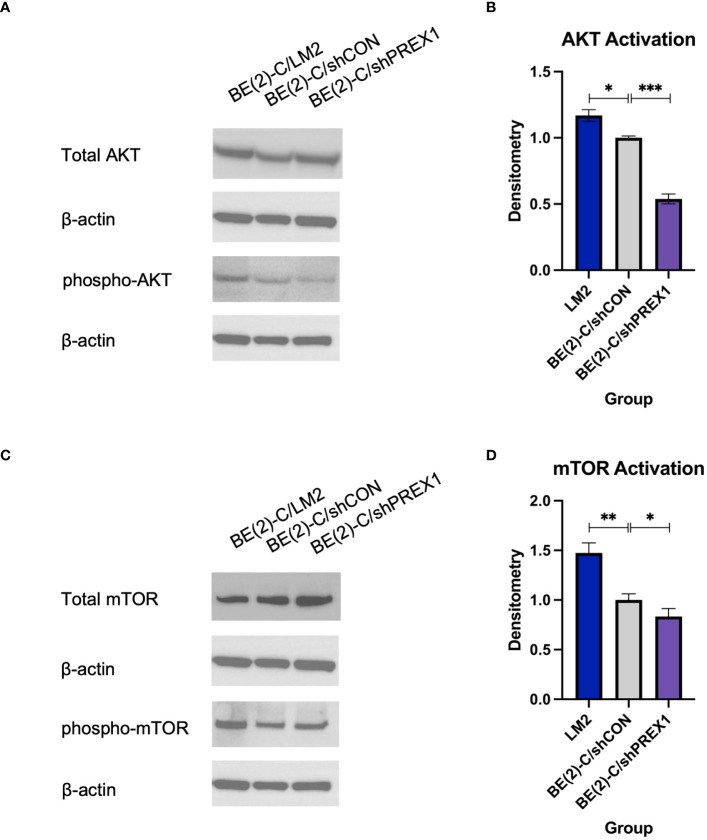
Silencing of *P-Rex1* decreased activity of the AKT-mTOR pathway. **(A)** BE(2)-C/shPREX1 cells demonstrated decreased activation of AKT relative to control, whereas LM2 cells demonstrated increased activation. **(B)** Densitometry analysis of AKT activity was performed, analyzed as a ratio of the density of each protein band relative to the density of each housekeeping control band, and then presented as a ratio of phosphorylated AKT protein expression to total AKT protein expression to represent AKT activity. **(C)** BE(2)-C/shPREX1 cells demonstrated decreased activation of mTOR relative to control, whereas LM2 cells demonstrated increased activation. **(D)** Densitometry analysis of mTOR activity was performed, analyzed as a ratio of the density of each protein band relative to the density of each housekeeping control band, and then presented as a ratio of phosphorylated mTOR protein expression to total mTOR protein expression to represent mTOR activity. (mean ± SEM; ns, not significant; * = *p* < 0.05; ** = *p* < 0.01; ***p < 0.001).

*P-Rex1* is modulated by the activity of *PI3K* (12, 16, 29). Activation of *PI3K* stimulates *P-Rex1*, which is thought to subsequently have downstream effects on *AKT* and *mTOR* (29). Given *AKT* and *mTOR*’s known role in cancer cell migration (12), immunoblotting was used to evaluate whether *P-Rex1* affects AKT and mTOR activity in neuroblastoma. BE(2)-C/shPREX1 cells demonstrated decreased activation of both AKT (**Figures 5A, B**) and mTOR (**Figures 5C, D**) relative to control, whereas LM2 cells demonstrated increased activation. Utilizing densitometry, this decrease in activation associated with silencing of *P-Rex1* was particularly profound for AKT, as expressed by a ratio of phosphorylated protein expression to total protein expression. The BE(2)-C/shPREX1 cell line demonstrated approximately half the AKT activation of the BE(2)-C/shCON cell line (mean AKT activation for BE(2)-C/shCON: [normalized to] 1 ± 0.008 vs. BE(2)-C/shPREX1: 0.539 ± 0.021, *p* = 0.0007).

### 3.8 *P-Rex1* expression was correlated with and modulated by *Rac2*, as opposed to *Rac1* or *Rac3*

The publicly available Kocak database, comprised of 649 human neuroblastoma samples, was accessed to evaluate for associations between expression of *P-Rex1* and the three Rac effectors: *Rac1*, *Rac2*, and *Rac3. Rac1* is ubiquitously expressed in mammalian tissue and the most extensively studied Rac effector (30). *Rac2* is only expressed in hematopoietic and endothelial cells (30, 31). *Rac3* is highly expressed in the brain and has been implicated in neuronal development and tumor progression (30, 32, 33).

**Figure 6:**
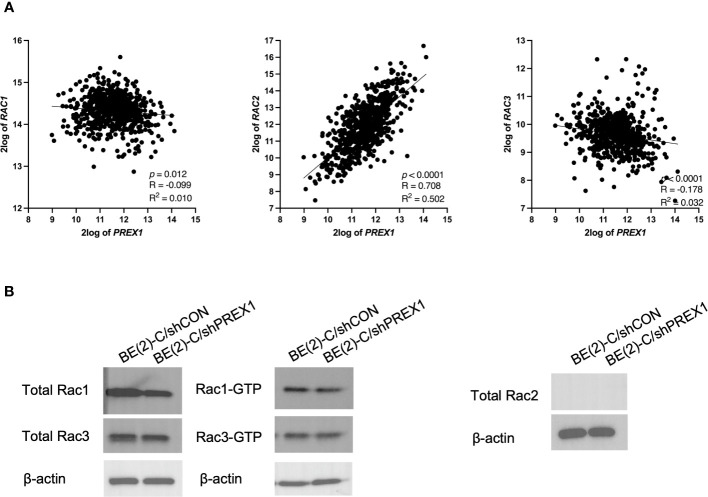
*P-Rex1* expression was correlated with and modulated by *Rac2*, as opposed to *Rac1* or *Rac3*. The publicly available Kocak database was accessed to evaluate for associations between expression of *P-Rex1* and the three Rac effectors: *Rac1*, *Rac2*, and *Rac3.* **(A)** Whereas *Rac1* and *Rac3* had statistically significant but relatively weak negative associations with expression of *P-Rex1*, *Rac2* had a robust, significant, and positive correlation with expression of *P-Rex1*. **(B)** On Rac GTPase assays, the BE(2)-C/shPREX1 cell line did not demonstrate any appreciable decrease in activation of Rac1 or Rac3 protein relative to BE(2)-C/shCON. Neither neuroblastoma cell line produced any total Rac2 protein.

Whereas expression of *Rac1* and *Rac3* had statistically significant but relatively weak negative associations with expression of *P-Rex1* (*P-Rex1* vs. *Rac1*: *p* = 0.012, R = -0.099, R^2^ = 0.010; *P-Rex1* vs. *Rac3*: *p* < 0.0001, R = -0.178, R^2^ = 0.032), *Rac2* had a robust, significant, and positive correlation with expression of *P-Rex1* (*P-Rex1* vs. *Rac2*: *p* < 0.0001, R = 0.708, R^2^ = 0.502) (**Figure 6A**).

Therefore, activation of Rac1, 2, and 3 were subsequently evaluated using Rac GTPase assays (**Figure 6B**). Consistent with the previously described genomics data, the BE(2)-C/shPREX1 cell line did not demonstrate any appreciable decrease in activation of Rac1 or Rac3 relative to BE(2)-C/shCON. Neither the BE(2)-C/shCON cells nor the BE(2)-C/shPREX1 cells produced any total Rac2 protein. This is consistent with data suggesting that Rac2 is specifically produced by hematopoietic cells (30, 31), and suggests an interplay between neuroblastoma cells and their surrounding tumor microenvironment in modulating *Rac2* expression within tumors.

### 3.9 Correlative expressions of *P-Rex1* and *ERK2/p42*

**Figure 7:**
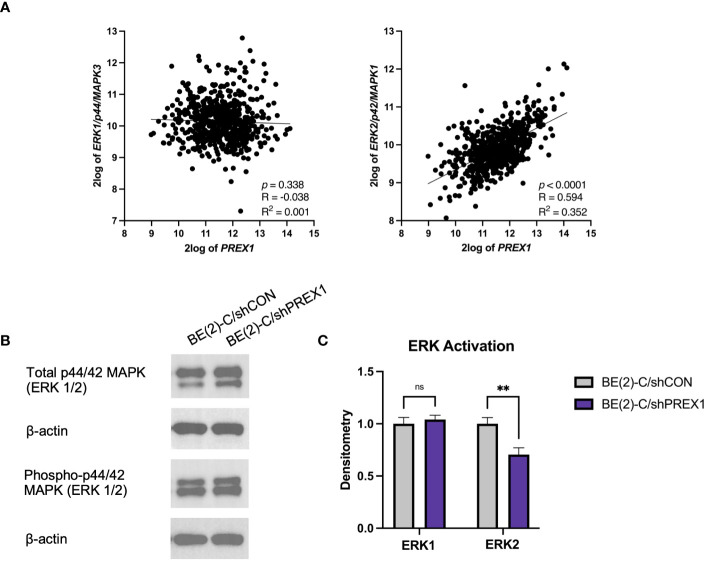
Expression of *P-Rex1* correlated with *ERK2/p42* expression. **(A)** The Kocak database was queried to assess for associations between expression of *P-Rex1* and *ERK1 and 2*. There was no significant association between the expression of *P-Rex1 and ERK1/p44*. However, there was a significant positive association between the expression of *P-Rex1* and *ERK2/p42*. **(B)** Immunoblotting demonstrating total and phosphorylated ERK1 and ERK2 protein expression by cell line. **(C)** Densitometry analysis of ERK1/2 activity was performed, analyzed as a ratio of the density of each protein band relative to the density of each housekeeping control band, and then presented as a ratio of phosphorylated ERK1/2 protein expression to total ERK1/2 protein expression to represent ERK activity. There was no significant difference in ERK1 protein activity between BE(2)-C/shCON and BE(2)-C/shPREX1 cell lines. However, there was a significant decrease in the activity of ERK2 protein associated with silencing of *P-Rex1*. (mean ± SEM; ns, not significant; ** = *p* < 0.01).

*P-Rex1* and *Rac* have been implicated in modulating *ERK1/2* signaling pathways (12, 18, 29, 34). The Kocak database was queried to assess for associations between the expression of *P-Rex1* and *ERK1* and *ERK2* (**Figure 7A**). There was no significant association between the expression of *P-Rex1 and ERK1/p44*. However, there was a significant positive association between the expression of *P-Rex1* and *ERK2/p42* (*p* < 0.0001, R = 0.594, R^2^ = 0.352). Results of immunoblotting evaluating levels of ERK protein activity in each cell line were consistent with these findings (**Figure 7B**). ERK activity was expressed as a ratio of phosphorylated ERK protein expression to total ERK protein expression using densitometry. There was no significant difference in ERK1 protein activity between BE(2)-C/shCON and BE(2)-C/shPREX1 cell lines. However, there was a significant decrease in the activity of ERK2 protein associated with silencing of *P-Rex1* (BE(2)-C/shCON: (normalized to) 1 ± 0.059 vs. BE(2)-C/shPREX1 0.706 ± 0.064, *p* = 0.004) (**Figure 7C**).

### 3.10 *P-Rex1* silencing was associated with decreased secretion of matrix metalloproteinases

**Figure 8:**
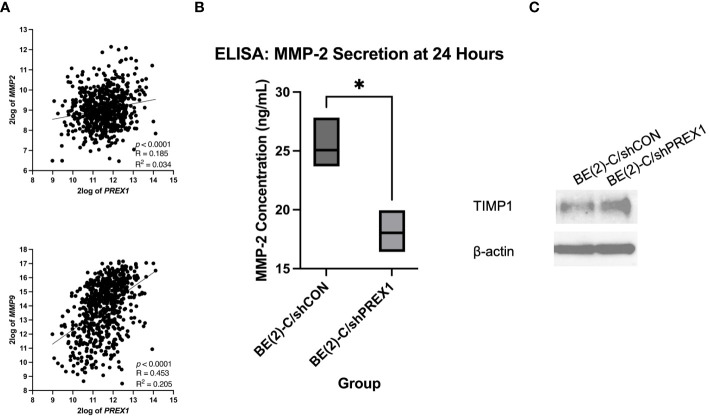
Silencing of *P-Rex1* decreased secretion of matrix metalloproteinases (MMPs) and increased protein expression of tissue inhibitors of metalloproteinases (TIMPs). **(A)** The Kocak database was queried to evaluate for a potential association between the expression of *P-Rex1* with *MMP-2* and *MMP-9*. *P-Rex1* expression was significantly and positively associated with both *MMP-2* and *MMP-9* expression. **(B)** BE(2)-C/shPREX1 cells demonstrated significantly decreased secretion of MMP-2 at 24 hours relative to control on ELISA. **(C)** BE(2)-C/shPREX1 cells demonstrated increased protein expression of TIMP1 on immunoblotting relative to control. * = *p* < 0.05.

MMPs are enzymes capable of degrading extracellular matrix proteins (35). MMP-2 and MMP-9, in particular, have been associated with cancer metastasis (35). The Kocak database was queried to evaluate for a potential association between the expression of *P-Rex1* with *MMP-2* and *MMP-9* (**Figure 8A**). *P-Rex1* expression was significantly and positively associated with both *MMP-2* (*p* < 0.0001, R = 0.185, R^2^ = 0.034) and *MMP-9* (*p* < 0.0001, R = 0.453, R^2^ = 0.205) expression. Increased expression of MMPs at the mRNA and protein level has been associated with poor prognosis in numerous cancers (35).

To further investigate the mechanism by which *P-Rex1* affects neuroblastoma migration and metastasis, ELISA was performed to evaluate *P-Rex1*’s effect on the secretion of MMPs *in vitro*. MMP-2 is secreted by both neuroblastic tumor cells as well as stromal cells (36, 37). BE(2)-C/shPREX1 cells demonstrated significantly decreased secretion of MMP-2 at 24 hours relative to control (18.0 ± 1.0 ng/mL vs. 25.0 ± 1.4, *p* = 0.018) (**Figure 8B**). MMP-9, however, is exclusively expressed by stromal cells (36, 37). These results were confirmed after ELISA demonstrated a concentration of zero for MMP-9 on analysis of both BE(2)-C/shCON and BE(2)-C/shPREX1 neuroblastoma cells. Previous studies have found that neoplastic cells can express factors that stimulate MMP secretion by stromal cells (37–39) and further work is needed to determine whether *P-Rex1* expression affects MMP-9 secretion by stromal cells in neuroblastoma.

### 3.11 Silencing of *P-Rex1* was associated with increased protein expression of tissue inhibitors of metalloproteinases

TIMPs are endogenous inhibitors of MMPs, and dynamic changes in the expression of MMPs and TIMPs have been associated with tumor metastasis (40). BE(2)-C/shPREX1 cells demonstrated increased protein expression of TIMP1 on immunoblotting relative to control (**Figure 8C**). These results suggest that the decreased secretion of MMPs may be in the setting of increased TIMP expression and subsequent inhibitory effect on MMPs.

### 3.12 Increased expression of *P-Rex1* was associated with worse clinical outcomes

**Figure 9:**
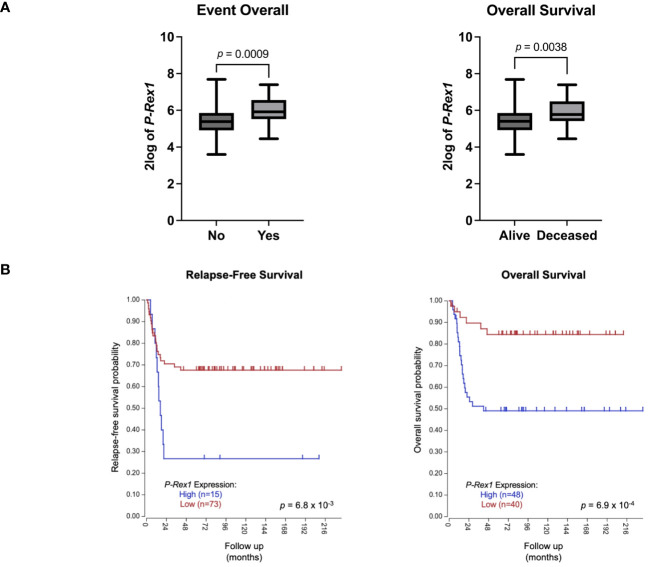
Increased *P-Rex1* expression was associated with worse clinical outcomes in the Versteeg clinical database. **(A)** Higher mean 2log expression of *P-Rex1* was seen in neuroblastoma patients who had events or died. **(B)** Increased expression of *P-Rex1* was associated with inferior relapse-free and overall survival on Kaplan-Meier analysis.

The publicly accessible Versteeg clinical database, available through the R2: Genomics Analysis and Visualization Platform (27), was utilized to evaluate for a possible association between *P-Rex1* expression and clinical outcomes. Higher mean 2log expression of *P-Rex1* was seen in neuroblastoma patients who had events (5.965 vs. 5.381, *p* = 0.0009) or died (5.895 vs. 5.397, *p* = 0.0038) (**Figure 9A**). Furthermore, increased expression of *P-Rex1* was associated with inferior relapse-free and overall survival on Kaplan-Meier analysis (*p = 6.8 x 10^–3^ and p = 6.9 x 10^–4^*, respectively) (**Figure 9B**).

## 4 Discussion

**Figure 10:**
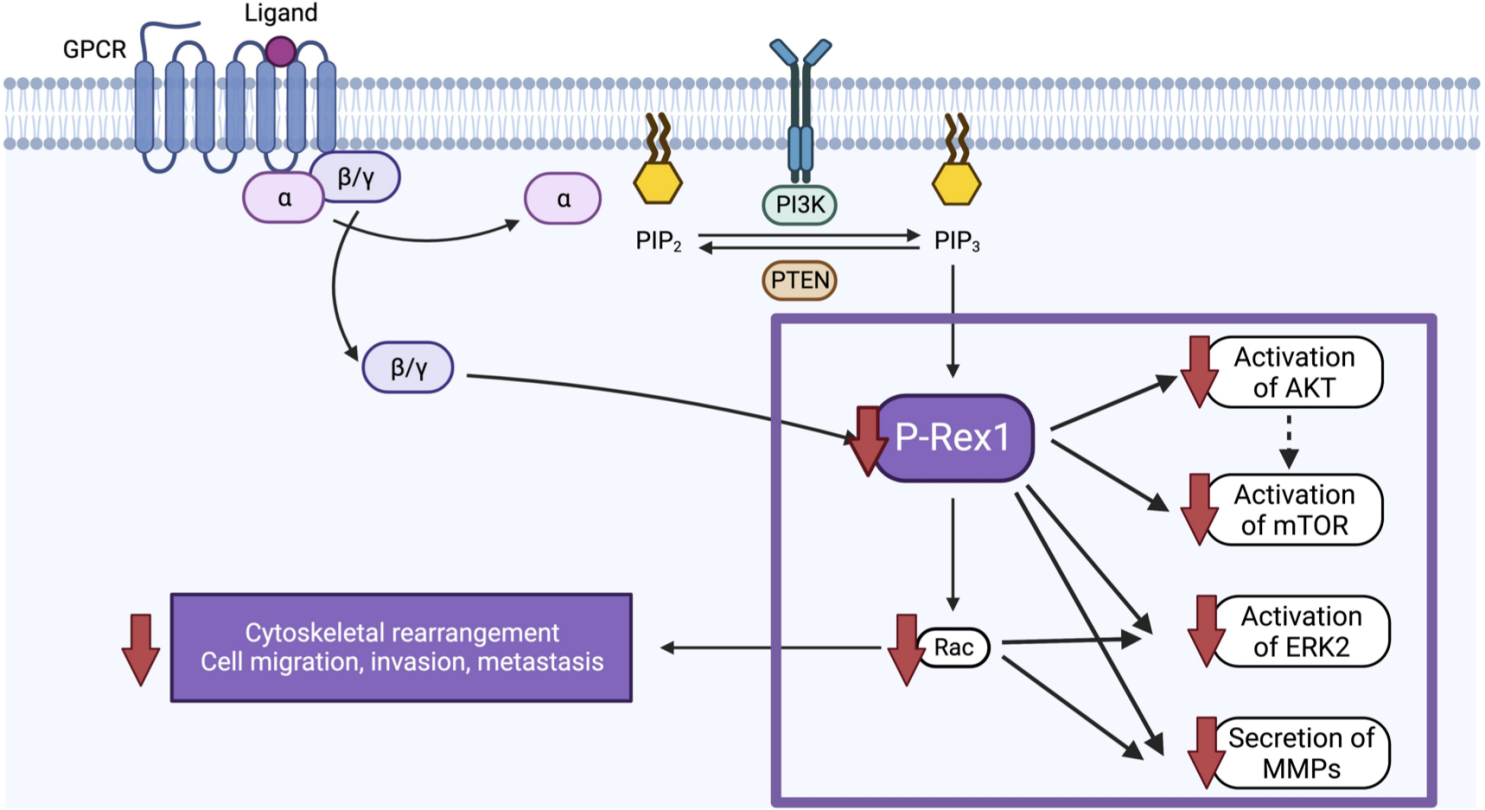
Schematic of the proposed function and mechanism of *P-Rex1* in neuroblastoma. Image created with BioRender.com.

Despite intensive multimodal therapy, children with high-risk neuroblastoma continue to demonstrate significant morbidity and mortality due, in part, to the high rates of metastatic disease found at diagnosis. A better understanding of the biological factors that drive migration, invasion, and metastasis in neuroblastoma is needed to develop novel therapies that specifically target this aggressive disease phenotype. In the present study, we found that pro-metastatic murine models of neuroblastoma and human neuroblastoma metastases demonstrate upregulation of P-Rex1. Correspondingly, silencing of *P-Rex1* decreased migration and invasion of neuroblastoma cells *in vitro*. This is likely due to decreased activation of the AKT-mTOR pathway, dysregulation of the Rac effector (predominantly Rac2), downregulation of ERK2 activity, and diminished secretion of MMPs (**Figure 10**). Furthermore, increased expression of *P-Rex1* was associated with inferior relapse-free and overall survival on Kaplan-Meier analysis of the publicly accessible Versteeg clinical database (27). This suggests that *P-Rex1* may be a novel therapeutic target and prognostic marker in neuroblastoma.

In preclinical models of other cancer types, *P-Rex1* has been found to have numerous and variable functions. In the present study, *P-Rex1* modulated migration, invasion, and metastasis but demonstrated negligible effects on primary neuroblastoma cell viability. Our findings are consistent with several studies evaluating the role of *P-Rex1* in melanoma and prostate cancer (13, 17, 41). *P-Rex1* deficiency was associated with impaired migration, invasion, and metastasis in mouse models of melanoma and prostate cancer; however, there was no significant reduction in proliferation or primary tumor burden (13, 17, 41). Our results differ from many studies of breast cancer and liver cancer in which *P-Rex1* also affected tumor initiation and proliferation of the primary tumor (15, 18, 29, 34). Furthermore, in this study, the primary Rac effector of neuroblastoma appeared to be Rac2, as opposed to Rac1 in melanoma, prostate cancer, breast cancer, and liver cancer (13, 15, 18, 29, 34, 41, 42). In all previously mentioned cancer types, including neuroblastoma in the present study, however, P-Rex1 demonstrated negligible expression on IHC in benign tissue, with highest expression at sites of tumor invasion and metastasis relative to primary tumor sites (13, 15–17, 41, 43). These IHC results suggest that P-Rex1 facilitates metastatic dissemination of cancer cells and/or is upregulated after metastasis (12). Collectively, our findings indicate that although *P-Rex1* modulates migration, invasion, and metastasis ubiquitously across cancer types, it may have variable or context-dependent effects on other cellular functions as well as divergent mechanisms across malignancies.

The results of the present study have substantial potential translational relevance. First, inhibition of P-Rex-Rac GEFs is an attractive therapeutic strategy given their key roles in oncogenesis but dispensable nature in developmental processes (11). Furthermore, given that P-Rex1 expression is negligible in benign or normal tissues but overexpressed or mutated in many human cancers, particularly at sites of metastasis (12, 13, 15–17, 41, 43), it has the potential to be a more specific therapeutic target that spares normal tissue and therefore mitigates overall treatment morbidity. This also makes it a potentially more advantageous target for inhibition than *PI3K* or *Rac*, for example, given their more ubiquitous expression and subsequently increased likelihood of inadvertent secondary effects. Moreover, *P-Rex1* has been found to promote resistance to other therapies commonly used in practice. Goel et al. found that induction of *P-Rex1* promoted Rac1-mediated ERK activation that was associated with resistance to anti-vascular endothelial growth factor (VEGF)/anti-vascular endothelial growth factor receptor (VEGFR) therapies in prostate cancer (44). The authors suggested that combination P-Rex1-Rac inhibition with other currently utilized inhibitory therapeutic strategies, such as anti-angiogenesis agents, may improve the latter’s clinical efficacy. Finally, inhibition of *P-Rex1* may be a particularly advantageous novel therapeutic strategy in neuroblastoma given its association with the *Rac2* effector. Although predominantly studied in the setting of hematologic pathophysiology, in studies of solid tumors, *Rac2* has demonstrated a critical role in macrophage differentiation and the metastatic phenotype. Using syngeneic and orthotopic tumor models, Joshi et al. found that Rac2-/- mice demonstrated significant defects in tumor growth and metastasis (31). In addition, Rac2 activity was required for polarization of macrophages to an anti-inflammatory, pro-tumorigenic phenotype (31). These results have significant clinical ramifications, particularly in neuroblastoma. Unlike in other cancer types, pediatric patients with metastatic neuroblastoma typically undergo surgical resection due to studies demonstrating improvements in event-free survival in patients with high-risk neuroblastoma who undergo ≥ 90% resection of their primary tumor (45). However, primary tumor resection has been implicated in surgery-accelerated metastasis in several cancers (46–48) via polarization of tumor-associated macrophages into a pro-tumorigenic phenotype (47). Therefore, inhibition of *P-Rex1*, which is directly upstream of *Rac*, has the potential to mitigate the metastasis seen in standard oncogenesis, as well as surgery-accelerated metastasis. Additionally, although systemic therapy is typically suspended during the perioperative period due to concerns for wound healing, P-Rex1 inhibitors could potentially be considered and tested as a novel perioperative therapy given the low expression of P-Rex1 in normal tissue. Together, these results suggest that *P-Rex1* inhibition has numerous potential benefits in the therapeutic management of neuroblastoma.

Although small molecule inhibitors of *P-Rex1* are currently being developed (11, 49), further work is required for phase I trials. Also, inhibition of *P-Rex1* may result in the regulation of other genes and proteins that may affect neuroblastoma tumorigenesis. Furthermore, additional study is warranted evaluating the role of *P-Rex1* in additional cell lines and on modulation of the tumor microenvironment and stroma, including regulation of Rac2 and subsequent effects on macrophage differentiation (31). In addition, the lack of observed Rac1 and Rac3 binding seen in the present study may reflect minimal regulation of these Racs by *P-Rex1* relative to the overall Rac1 or 3 activity levels within neuroblastoma cells and warrants further investigation. Nonetheless, our results demonstrate an important role of *P-Rex1* in neuroblastoma cell migration, invasion, and metastasis.

In conclusion, migration, invasion, and metastasis are mediated by *P-Rex1* in neuroblastoma. The cellular mechanisms responsible for this phenomenon appear to be multifaceted and involve modulation of the AKT-mTOR and ERK signaling pathways, dysregulation of *Rac*, and altered secretion of MMPs. Inhibition of *P-Rex1* may be an efficacious, novel therapeutic strategy in the management of neuroblastoma, and in particular, tumors with aggressive, pro-metastatic phenotypes. Furthermore, given that increased *P-Rex1* expression was associated with inferior clinical outcomes, it may also serve as a novel prognostic factor. To our knowledge, this is the first study evaluating the role of *P-Rex1* in neuroblastoma (or any solid tumor predominantly diagnosed in children) and describing its potential as a novel therapeutic management strategy in neuroblastic tumors.

## Data availability statement

The original contributions presented in the study are included in the article/**Supplementary Material**, further inquiries can be directed to the corresponding author/s.

## Ethics statement

The requirement of ethical approval was waived by UT Southwestern Human Research Protection Program for the studies on humans because the human tissue samples were de-identified and did not affect patient care. The studies were conducted in accordance with the local legislation and institutional requirements. Written informed consent for participation was not required from the participants or the participants’ legal guardians/next of kin in accordance with the national legislation and institutional requirements. The human samples used in this study were acquired from the UT Southwestern/Children’s Medical Center Dallas Department of Pathology, who had formalin-fixed, paraffin-embedded, and archived them with informed consent for research purposes. The animal study was approved by Institutional Animal Care and Use Committee at Vanderbilt University Medical Center. The study was conducted in accordance with the local legislation and institutional requirements.

## Author contributions

JJ: Conceptualization, Data curation, Formal analysis, Funding acquisition, Investigation, Methodology, Validation, Visualization, Writing – original draft, Writing – review & editing. JQ: Conceptualization, Data curation, Formal analysis, Investigation, Methodology, Resources, Validation, Visualization, Writing – original draft, Writing – review & editing. EC: Writing – review & editing. SM: Data curation, Writing – review & editing. DC: Conceptualization, Formal analysis, Funding acquisition, Investigation, Project administration, Resources, Supervision, Writing – review & editing.

## Supplementary material

Supplementary Figure 1 Silencing of *P-Rex1* decreased invasion of JF neuroblastoma cells. After 96 hours of incubation, invaded JF/siCON cells demonstrated colony formation whereas JF/siPREX1 cells failed to do so, further demonstrating inhibition of invasion associated with silencing of *P-Rex1.*
