# Supplementary figures and images for: Migration, invasion, and metastasis are mediated by P-Rex1 in neuroblastoma
Source: Front Oncol. 2024 May 31;14:1336031. doi: 10.3389/fonc.2024.1336031 (PMC11176429; doi:10.3389/fonc.2024.1336031)

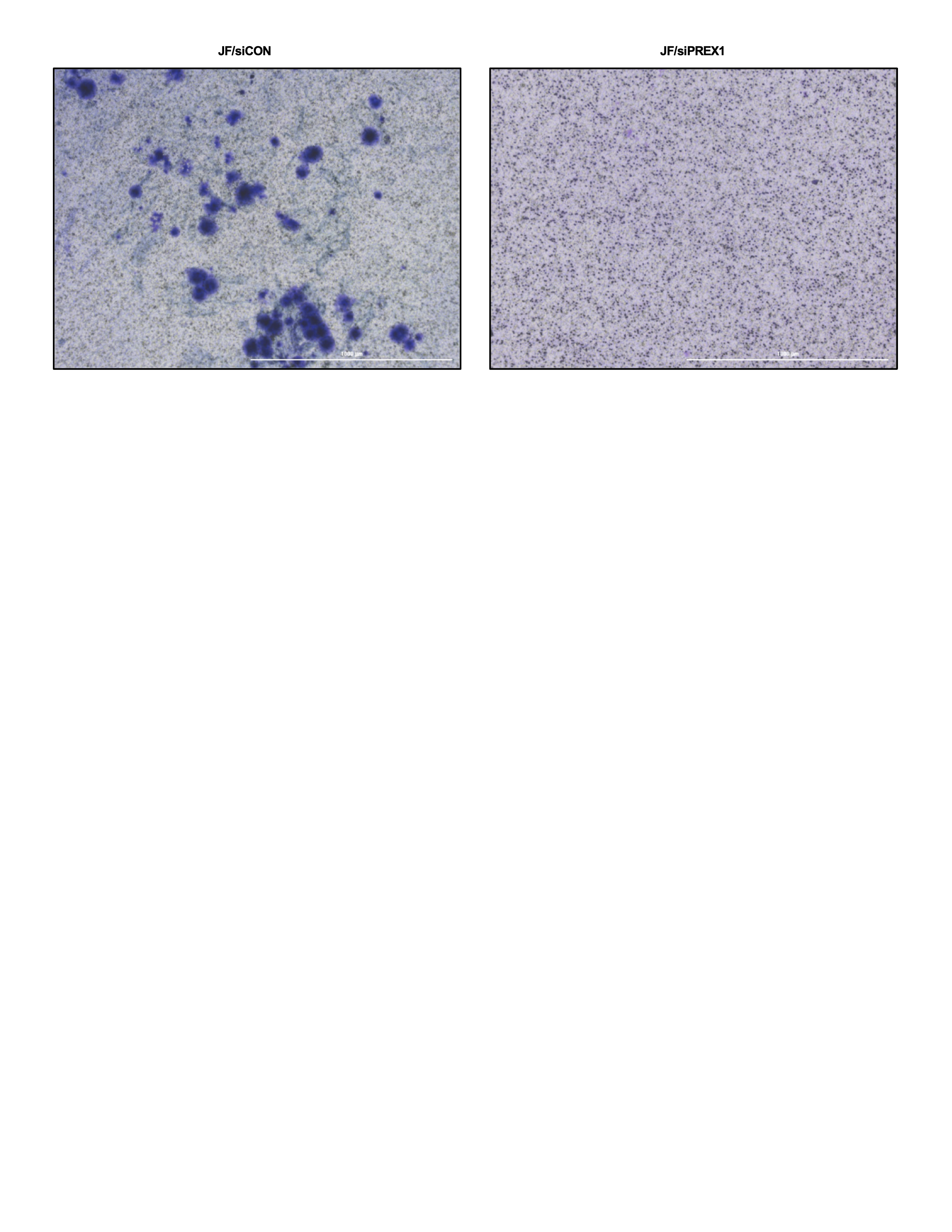

Supplement: Supplementary Figure 1 — Silencing of P-Rex1 decreased invasion of JF neuroblastoma cells. After 96 hours of incubation, invaded JF/siCON cells demonstrated colony formation whereas JF/siPREX1 cells failed to do so, further demonstrating inhibition of invasion associated with silencing of P-Rex1. [file Image_1.tiff]
